# Non-Gaussian Diffusion Imaging for Enhanced Contrast of Brain Tissue Affected by Ischemic Stroke

**DOI:** 10.1371/journal.pone.0089225

**Published:** 2014-02-27

**Authors:** Farida Grinberg, Ezequiel Farrher, Luisa Ciobanu, Françoise Geffroy, Denis Le Bihan, N. Jon Shah

**Affiliations:** 1 Institute of Neuroscience and Medicine - 4, Forschungszentrum Juelich GmbH, Juelich, Germany; 2 NeuroSpin, Commissariat à l'énergie atomique et aux énergies alternatives (CEA Saclay), Gif-sur-Yvette, France; 3 Department of Neurology, Faculty of Medicine, JARA, RWTH Aachen University, Aachen, Germany; University of Minnesota, United States of America

## Abstract

Recent diffusion MRI studies of stroke in humans and animals have shown that the quantitative parameters characterising the degree of non-Gaussianity of the diffusion process are much more sensitive to ischemic changes than the *apparent diffusion coefficient* (ADC) considered so far as the “gold standard”. The observed changes exceeded that of the ADC by a remarkable factor of 2 to 3. These studies were based on the novel non-Gaussian methods, such as diffusion kurtosis imaging (DKI) and log-normal distribution function imaging (LNDFI). As shown in our previous work investigating the animal stroke model, a combined analysis using two methods, DKI and LNDFI provides valuable complimentary information. In the present work, we report the application of three non-Gaussian diffusion models to quantify the deviations from the Gaussian behaviour in stroke induced by transient middle cerebral artery occlusion in rat brains: the gamma-distribution function (GDF), the stretched exponential model (SEM), and the biexponential model. The main goal was to compare the sensitivity of various non-Gaussian metrics to ischemic changes and to investigate if a combined application of several models will provide added value in the assessment of stroke. We have shown that two models, GDF and SEM, exhibit a better performance than the conventional method and allow for a significantly enhanced visualization of lesions. Furthermore, we showed that valuable information regarding spatial properties of stroke lesions can be obtained. In particular, we observed a stratified cortex structure in the lesions that were well visible in the maps of the GDF and SEM metrics, but poorly distinguishable in the ADC-maps. Our results provided evidence that cortical layers tend to be differently affected by ischemic processes.

## Introduction

Diffusion magnetic resonance imaging (MRI) is known as an important tool in early diagnostics and assessment of stroke [1], [2]. Usually, the *apparent diffusion coefficient* (ADC) exhibits a strong reduction within the first half an hour after the onset of infarction and allows for a visualisation of the ischemic lesion prior to manifestation by other conventional MRI modalities. Diffusion changes are associated with a failure of the sodium/potassium pump, giving rise to an inter-compartmental water shift and cell swelling (cytotoxic oedema). In spite of the high clinical relevance and intensive studies, the biophysical mechanisms of the observed ADC reduction are not yet well understood [3]. They are most frequently ascribed to the combined effects of restricting more water in swollen cells and an increased tortuosity of the extracellular space. Additional mechanisms may include changes in membrane permeability [4], amount of bound water [5], destruction of intracellular organelles [6] and cytoplasmic streaming [7]. More recent studies suggest focal enlargements of cellular projections (the so-called neurite beading) [8]–[10] as an essential mechanism of decreasing the diffusion coefficient.

Most of the reported diffusion stroke studies were performed in the low range of diffusion-weightings (*b*-value), ≤1 µm^−2^ ms, typical for conventional diffusion MRI. In this range, the diffusion-weighted (DW) signal of water in the brain tissue as a function of the *b*-value can be approximated by a monoexponential function, under the assumption of a Gaussian diffusion propagator [11]. For larger *b*-values, the deviations from the monoexponential function become significant [12]–[14] and a concept of the ADC [15] becomes insufficient to describe the signal attenuation curve. The exact mechanisms underlying these deviations are not yet fully elaborated. Generally, they are attributed to various motional restrictions and barriers imposed by tissue microstructure, such as cellular membranes or myelin, and may involve complex interactions with cellular proteins and membrane permeability. Due to the enormous complexity of tissue organisation, various contributions to the measured DW signal cannot be decomposed easily. However, as recently emphasized [16], it is imperative to employ higher *b*-values in order to get a better access to the intracellular space and membrane interactions and thus render the DW signal more representative of the cellular microstructure than surrounding extracellular water.

In recent years, non-Gaussian diffusion methods permitting the analysis of the DW signal over a larger range of *b*-values have gained an increasing importance in brain research. During the last one and a half decades several approaches, such as diffusion kurtosis imaging (DKI) [17], [18], biexponential diffusion tensor analysis (BEDTA) [13], [14], [19]–[21], the statistical model by Yablonskiy [22], the stretched-exponential model (SEM) [12], [23] or its modifications to describe anomalous diffusion [24]–[28], have been suggested to account for deviations from the Gaussian model. Although a link with tissue microscopic features is not straightforward, all these approaches permit the quantification of the degree of non-monoexponentiality of the DW signal based on different assumptions [29], [30]. Some approaches, such as DKI, are empirical in their nature and aim to describe the attenuation of the MRI signal using a set of phenomenological parameters. Other models use geometric schemes [31]–[33], such as a set of oriented cylinders describing axonal formations in white matter (WM), or a set of macroscopically disordered cylinders as an approximate of neuronal processes in gray matter (GM). In particular, DKI is a model-free extension of conventional diffusion MRI (in particular, diffusion tensor imaging, DTI), making use of a higher order quadratic term in the Taylor series expansion of the natural logarithm of the DW signal. BEDTA represents the simplest realization of the frequency distribution of the ADCs given by a discrete sum of two exponentials. It assumes the existence of two water pools with different ADCs. These pools can be attributed, for example, to different compartments (intracellular, ICS, and extracellular, ECS, space) [32], or to free and bound water [5]. The statistical model [22] extends a discrete number of pools to a continuous distribution of ADCs. In this way it accounts for the complexity and heterogeneity of the tissue microstructure that imposes motional restrictions and hindrances on various length scales and models different local diffusion environments for diffusion of different spin ensembles. Further models specify axonal sizes and densities as a source of the ADC distribution and support the experimental protocols for their determination [34], [35].

These models provide a more accurate approximation of the DW signal than conventional diffusion MRI in the moderate to high *b*-value ranges [36] and enrich the information on the brain tissue microstructure [37], [38]. Nevertheless, regarding their applications to the study of diseases, non-Gaussian approaches are still at an early stage since the related progress remained rather slow, mainly, due to technical problems. In the early implementations, the impediment to clinical adoption was primarily due to a severe decrease in the signal-to-noise ratio with increasing *b*-values, leading to a prolongation of the acquisition time above the clinical requirements. In recent years, however, more and more applications have been reported to provide enhanced information on microstructural properties of healthy and pathological tissues. A real breakthrough was achieved with DKI in recent years as it has been demonstrated to provide promising biomarkers in healthy aging [39], [40], stroke [9], [10], [41]–[43] and in neurodegenerative diseases, such as Parkinson's [44], Huntington's [45], and Alzheimer's diseases [46]. Besides, DKI has been demonstrated to be helpful in glioma grading [47], [48]. Promising applications of the SEM have been reported by Bennett et al. to investigate brain tumours [49], and also BEDTA has been shown to provide interesting results in the study of tumours [49], [50].

Regarding stroke, an enhancement of DKI metrics in ischemic lesions was observed by Jensen et al. [10] in humans and a similar enhancement was reported in the animal stroke model by Grinberg et al. [41]. Interestingly, the amount of change in DKI parameters reported in these two works was very similar in spite of the differences in the substrate tissues carrying the lesions, that is, WM in the human brain [10] and GM in the rat brain [41]. In both tissue types, the ADC has changed by 30–40%, typical for stroke, whereas the mean kurtosis (MK) changed up to 100–150% within the first 24 hours after symptom onset. A larger absolute percent change in kurtosis metrics in comparison to the ADC was also reported by Hui et al. [9] for a large group of stroke patients (retrospective study of 44 patients) who underwent investigations within the first 2 weeks after the onset. Based on a WM model that describes the microstructure in terms of extra- and intra-axonal environments, the observed changes in diffusion metrics were attributed to a significant drop in the intra-axonal diffusion microenvironment as a dominating factor, consistent with a proposed mechanism of axonal beading [8].

In our previous work [41], the DKI study of the animal stroke model was complemented by the investigation of another non-Gaussian model called log-normal distribution function imaging (LNDFI). It is based on the statistical approach and describes the diffusion behaviour in terms of the log-normal distribution function of ADCs. It was shown to provide a good signal description of the DW signal down to 4% of the initial non-DW value. Previous results have demonstrated that the peak diffusivity, *D*_LD_, and the scale parameter, *σ*_LD_, of the LNDFI model exhibit an enhanced sensitivity to ischemic lesions, i.e. ∼60% change in *σ*_LD_ and ∼50% in *D*_LD_.

These findings raised our interest in the following question: what are the advantages of other non-Gaussian models suggested in the literature and how they will perform in the assessment of stroke in comparison to each other? In this work we provide a characterisation of the ischemic lesions in animals by three non-Gaussian models: a) SEM, b) the statistical model based on the gamma distribution function (GDF), and c) BEDTA. Our goal was to investigate the applicability and sensitivity of the quantitative metrics in these models as biomarkers of stroke-induced microstructural changes in animals. We compared the performance of these models with previously investigated DKI and LNDFI. We hypothesized that simultaneous application of several models can provide added value in the assessment of stroke.

## Theoretical Background

### Monoexponential and kurtosis models

In isotropic non-confined media, diffusion is described by the Gaussian propagator. The conjunct normalised DW signal (i.e. the signal intensity divided by its value in absence of diffusion-weighting gradients), *S*(*b*), is given by a monoexponential function [51]

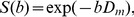
(1)where *D*_m_ is the diffusivity and *b* is the diffusion-weighting factor depending on the strength, duration, and separation intervals of the magnetic field gradient pulses. In isotropic ordinary liquids, Eq. (1) provides an estimate of the intrinsic diffusion coefficient determined merely by viscosity and temperature. In brain tissue, Eq. (1) provides a good approximation of the signal decay only for low *b*-values used in conventional diffusion MRI. However, the experimental values of *D*_m_ appear reduced with respect to the intrinsic diffusivity value due to the influence of various motional barriers (cellular membranes, myelin sheaths, organelles, etc.) and other interactions during the typical observation times. Moreover, in macroscopically anisotropic media, such as WM, the experimental values of *D*_m_ are reduced by an amount that depends also on the direction of the diffusion-weighting magnetic field gradients. Using the measurements of the DW signal in at least 6 gradient directions DTI describes the diffusion behaviour in terms of scalar metrics such as mean diffusivity (MD) or fractional anisotropy (FA) [52]. In complex media, the measured metrics will depend not only upon the genuine features of the underlying microstructure itself but also on the experimental parameters such as sequence timings. To keep these factors in mind, the diffusion coefficient evaluated from the pulsed field gradient MRI experiments in brain tissue is conventionally referred to as the ADC [15].

DKI accounts for deviations from the pattern of Gaussian diffusion by including a second-order term in the Taylor expansion of the natural logarithm of the DW signal. The DW signal then can be written as [17]

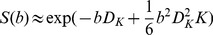
(2)where *D*_K_ and *K* denote the apparent diffusivity and the apparent kurtosis for an individual gradient direction, respectively. *D*_K_ determines the initial slope of the signal attenuation curve
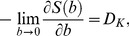
(3)and coincides with the ADC evaluated for the same gradient direction under the assumption of monoexponential approximation in the low *b*-value range. MK can be evaluated as the arithmetic average of *K* along various gradient directions [37]. Due to the truncation of higher order terms, applicability of DKI is limited to a moderate range of *b*-values, 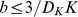.

### Statistical model

Generally, heterogeneous systems give rise to more than one single diffusion coefficient. Assuming a continuous distribution of diffusion coefficients, *P*(*D*), the DW signal can be written as follows
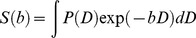
(4)with the initial slope of the attenuation curve yielding the mean diffusivity, 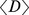, of the distribution
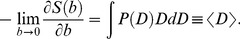
(5)


A comparison with the monoexponential model shows that 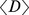 will be close to *D_m_* evaluated via Eq. (1) in the range of very low *b*-values. It should be noted that a similar differentiation between the intrinsic and reduced diffusion coefficients as in the case of monoexponential function applies for distributed diffusivities. As an example, the diffusivity distributions in polymer solutions are most frequently due to a distribution of macromolecular sizes. In that case, the measured distribution refers to intrinsic diffusivities and allows one to deduce information on polymer polydispersity [53], [54]. In contrast, the statistical model of diffusion in biological tissue [22] attributes the distribution to a large number of spin packets each characterised by its individual reduced (or apparent) diffusivity. The reason for the distribution is that, because of restrictions and hindrances in a disordered media, various spin packets tend to explore somewhat different local environments during the observation time. In general, the individual spin packets are due to heterogeneity of the environment and do not necessarily require an existence of separated physical compartments in the tissue. The distribution of reduced diffusivities due to motional barriers, giving rise to non-monoexponential signal attenuations, can originate also from the same compartment as was demonstrated, for example, by Monte Carlo simulations [55] and phantom studies [56] for diffusion in the interstitial space between oriented cylindrical objects.

According to Eq. (4), the distribution function *P*(*D*) can be found from the experimental data via inverse Laplace transform. However, the latter represents a well-known, ill-conditioned mathematical problem and leads to unstable computational solutions [57]. Therefore, for practical purposes, it is more convenient to assume an explicit form of *P*(*D*) and to fit it to the experimental values. In general, many functional forms may provide satisfactory fits to the experimental data points. The choice of such a function is usually done empirically since the related theories usually do not provide predictions for the specific shape of the distribution. The principal requirement is that the distribution function does not contain unphysical negative diffusivities and is thus asymmetric. Examples are the truncated normal distribution function [22] and the log-normal distribution function [58].

In the past, the log-normal distribution function was applied in the study of polymer diffusion [53] and, more recently, it has been used with the animal stroke model [41]. Being positive-definite it does not require an artificial cut off for negative diffusivity values. However, the disadvantage of this function is that, in combination with Eq. (4), it does not yield an analytically tractable integral and therefore requires time consuming numerical solutions. As an alternative, the gamma distribution function, *P*_G_(*D*) has been suggested recently and applied in the study of polymer diffusion [54]:
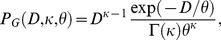
(6)where *Γ* is the gamma function, *θ* is the scale parameter of the same dimensionality as the diffusivity, and *κ* is the shape parameter. Replacing *P*(*D*) in Eq. (4) by *P*_G_(*D*), Eq. (6), gives the following expression for the DW signal attenuation [54]:
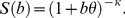
(7)The free parameters, *θ* and *κ*, can be expressed through the mean diffusion coefficient and standard deviation, σ_G_, as [54]:
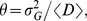
(8)

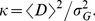
(9)In application to polydisperse polymers, Röding et al. [54] have shown that both functions, log-normal distribution function and GDF, provide strongly correlated estimates. However, the computational speed for GDF was much faster than for the log-normal distribution function making it a preferable choice in studies of macromolecular polydispersity.

### Biexponential model

The biexponential model represents a simple realisation of the distribution function with only two mode contributions
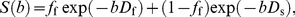
(10)where *D*_f_ and *D*_s_ are the (apparent) diffusivities of the fast and slow attenuation components, and *f*_f_ and 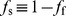 are the relative fractions of the fast and slow components, respectively. The biexponential function has been frequently evoked in the frame of “two-compartment” models [32], [59] for water in the extra-cellular and intra-cellular spaces, respectively. An alternative interpretation attributes the biexponential behaviour of the diffusion signal to the presence of “bound” and “free” water pools [5] that may exist within the same compartment. Statistically uniform but disordered media can also exhibit quasi-biexponential behaviour which then reflects the presence of distinct disorder length scales rather than the presence of separate compartments or pools [60]. Under certain circumstances, in the short time limit, a quasi-biexponential behaviour can be caused by the so-called “edge enhancement effects” due to restricting boundaries within the same compartment [61], [62]. In general, interpretation of the fast and slow diffusion processes in the brain tissue remains elusive [15] and requires caution in its application. Nevertheless, the biexponential function remains a useful model, especially in WM, as it captures significant features of the diffusion behaviour. BEDTA allows one to evaluate a single parameter, *α*_BEDTA_, quantifying the degree of non-monoexponentiality and sensitive to the presence of a slow diffusion fraction [14]:
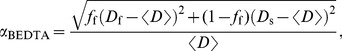
(11)where 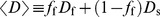. *α*_BEDTA_ = 0 corresponds to a monoexponential decay, which implies that either *D*_f_ = *D*_s_ = *D*_m_ or one of the component fractions is equal to 0 or to 1. In the opposite limit of the strongest non-monoexponentiality, *α*_BEDTA_ = 1, both fractions are equal to each other (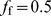) and one of the diffusivities is negligibly small (for instance, *D*_s_ = 0 µm^2^ ms^−1^).

### Stretched-exponential model

The stretched-exponential function is well known with regard to the global time relaxation behaviour of a disordered system that can be described by a superposition of independently relaxing exponential modes [63]. Bennett et al. [12] adapted this model to characterize heterogeneity of the DW signal as follows
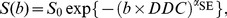
(12)where DDC is the distributed diffusion coefficient, and *α*_SE_ is the stretching exponent characterising deviations from the monoexponential behaviour (the so-called heterogeneity index, 0≤*α*_SE_≤1). For the Gaussian model, *α*_SE_ = 1. Note that the initial slope of the function does not provide the mean diffusivity, since the first derivative of Eq. (12) exhibits a singularity at *b* = 0. Obviously, the characteristic parameter of this model, DDC, does not coincide with the mean diffusivity except for *α*_SE_ = 1.

The stretched exponential function also arises in the context of a continuous time random walk model [64]. This model gives rise to anomalous diffusion behaviour by assuming that either jump lengths or time steps obey the power law distribution. In the anomalous and fractal model approaches [23], [25], [28], lower *α*_SE_ values indicate increasing disorder of the microenvironment in which the molecules diffuse.

## Materials and Methods

### Animals

Transient middle cerebral artery occlusion (MCAO) was induced [65] in three animals (300 g, Sprague-Dawley male rats) for the stroke experiments. The rats underwent a 90 min transient occlusion and were imaged 24 hours after reperfusion. The animals were anaesthetised with isoflurane (2%) administered in a mixture of air/oxygen through a nose cone and maintained at constant temperature (37°C) using a feedback-controlled air heating system (MR-compatible small animal heating system, SA Instruments, Inc., NY). The diffusion weighted acquisitions were respiration triggered. All experiments complied with French legislation and guidelines for animal research. The animal protocol was approved by the Comité d'EThique en Expérimentation Animale Commissariat à l'Energie Atomique et aux énergies alternatives Direction des Sciences du Vivant Ile de France (CETEA CEA DSV IdF).

### Immunohistochemistry and fluorescence microscopy

**Figure 1:**
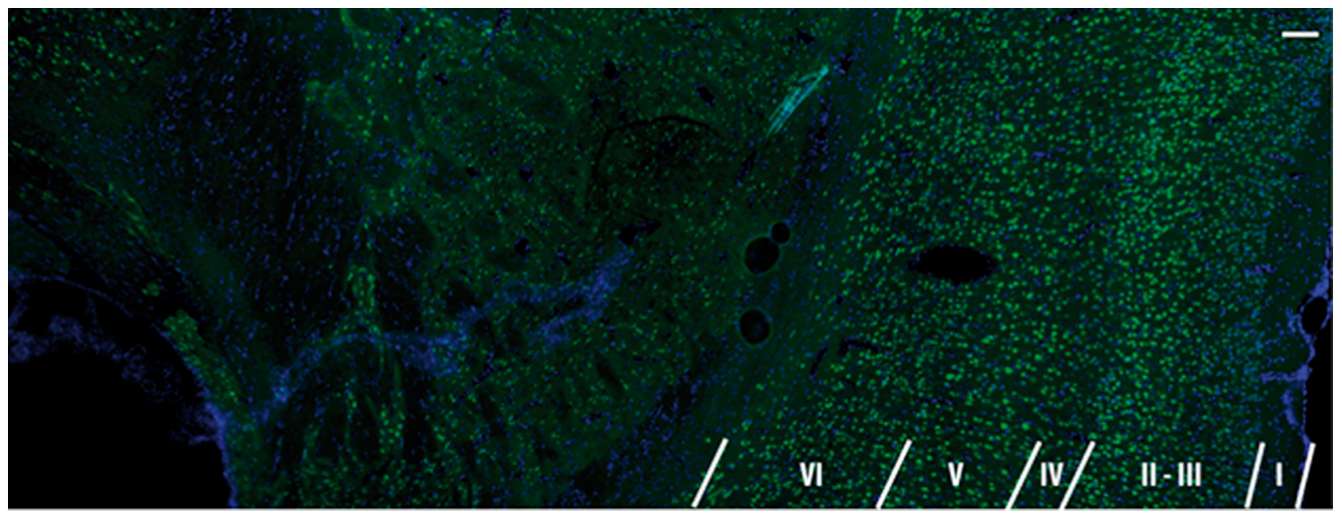
Photomicrograph showing the cortical lamination pattern in a normal rat brain. Superimposed photomicrograph of NeuN (antibody against neuronal nuclei), green, and DAPI (4′,6′-diamidino-2-phenylindole), blue, staining demonstrating the cortical lamination. Scale bar 100 µm.

Brains were perfused with a saline solution and, after complete blood removal, with 4% paraformaldehyde, and extracted. They were kept in paraformaldehyde for 2 h for tissue fixation, in 15% sucrose solution for 12 h and in 30% sucrose solution for 24 h for cryoprotection. 10 µm thick tissue slices were sectioned using a cryostat (Microm HM 560, Thermo Scientific, Courtaboeuf Cedex, France). Fluorescence microscopy was performed on an Axio Observer Z1 microscope (Carl Zeiss MicroImaging, Jena, Germany). One slice was treated with an antibody against neuronal nuclei (NeuN, Millipore, MAB377X, alexa 488 conjugated) and with 4′,6′-diamidino-2-phenylindole (DAPI) staining in order to highlight the different cell densities within the cortex. Figure 1 demonstrates NeuN/DAPI labeled photomicrograph showing the cortical lamination. Scale bar = 100 µm.

### MRI experiments

MRI experiments were performed on a 7T system (Bruker, PharmaScan) equipped with magnetic field gradients with maximum strength of 760 mT/m and a home-built RF surface coil (2.5 cm diameter). Rapid Acquisition with Refocused Echoes (RARE) T_2_-weighted images were acquired to localize the lesions (TR = 5530 ms, effective TE = 76 ms, FOV = 3×3 cm^2^, matrix size = 128×128, slice thickness = 1 mm, NEX = 2). Four-segment DW SE EPI images were acquired with the following acquisition parameters: TR/TE = 3000/30 ms, FOV = 3×3 cm^2^, matrix size 128×128, in-plane voxel size 234×234 µm^2^, slice thickness 1 mm, number of slices 4, slice gap 0.2 mm, diffusion field gradient duration *δ* = 5 ms, NEX = 4, and interpulse spacing between the gradient pulses *Δ* = 17 ms. The number of gradient directions was 20 (we omit a specification of the unit vectors since this investigation is focused mainly on the orientationally averaged parameters). The following five *b*-values were used for all animals: 0, 0.5, 1, 2.5, 3,5 µm^−2^ ms. Two more *b*-values at 5 and 6 µm^−2^ ms were added to the measurement protocol of animal 1 in order to produce a stronger attenuation and check its influence on the fitting procedure. In the following, the fitting ranges with *b*≤3.5 µm^−2^ ms and *b*≤6 µm^−2^ ms will be denoted as range 1 and range 2, respectively.

### Data analysis

Bias in the DW images due to the background noise was corrected using the power-images method [66], [67]. Computation of parameter maps was performed with in-house Matlab scripts (Matlab, The MathWorks, Natick, MA, USA). The normalized DW signal intensities, *S*(*b*), were fitted on a voxel-by-voxel basis using the functions described above, Eqs. (1, 2, 7, 10, 12), with the help of the nonlinear least-squares Nelder-Mead algorithm in the range of *b*-values indicated above, except for the monoexponential fits which were performed for *b*≤1 µm^−2^ ms. An upper boundary constraint (≤3 µm^2^ ms^−1^) was set according to the diffusivity of free water at 37°C.

The fits of the investigated models were compared for two *b*-value ranges (range 1 and range 2). The fit quality was quantified via evaluation of χ^2^- and R^2^-maps according to:
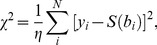
(13)

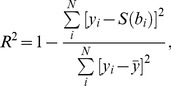
(14)where *y*_i_ is the normalised signal amplitude of the *i*-th experimental point, *S*(*b_i_*) is value of the fitting function, 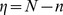 with *n* being the number of free parameters and *N* being the number of experimental points, 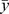 is the mean of the normalized signal amplitude. The mean absolute residuals were evaluated for each *b*-value as 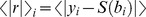, where the average is taken over all voxels in a region of interest (ROI) in all slices and all animals. χ^2^-maps were evaluated also for the monoexponential fits for different *b*-value ranges in order to visualize the deviations for increasing *b*.

The anisotropy of the DW signal in all ROIs was practically negligible since the ischemic lesions studied were located only in GM. Therefore, to enhance the SNR, the non-Gaussian models were fitted to the mean experimental curves averaged over all gradient directions. In the following, to appreciate the “apparent” nature of the evaluated diffusivities, we shall denote them as ADCs with subscripts indicating the model (“m” for monoexponential, “K” for DKI, “GD” for GDM, “LD” for LNDFI. Subscripts “f” and “s” will denote fast and slow diffusivities in BEDTA. The maps were constructed for DDC and *α*_SE_ (SEM), *θ* and *κ* (GDF), and ADC_f_, ADC_s_, and *f*_s_ (BEDTA). In addition, we evaluated 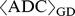 and *σ*_GD_ with Eqs. (8, 9) in order to enable a comparison with typical parameters such as the mean ADC and standard deviation. The histograms of the investigated parameters were taken over all slices for animal 1. The average values of different metrics were evaluated for two ROIs located in the ischemic lesions, and compared with the corresponding values in healthy counterparts. The ROIs were placed in the cerebral cortex (CT) and caudate putamen (CPu). The relative changes of the parameters in lesions compared to the healthy counterparts were evaluated, in per cent, as the ratio of the difference between the average values of a given parameter in the ischemic and healthy ROIs with respect to the average value in the healthy ROI. The averages were taken over all slices and animals (12 slices in total). Further details regarding the MR experiments and data processing are described elsewhere [41].

## Results

**Figure 2:**
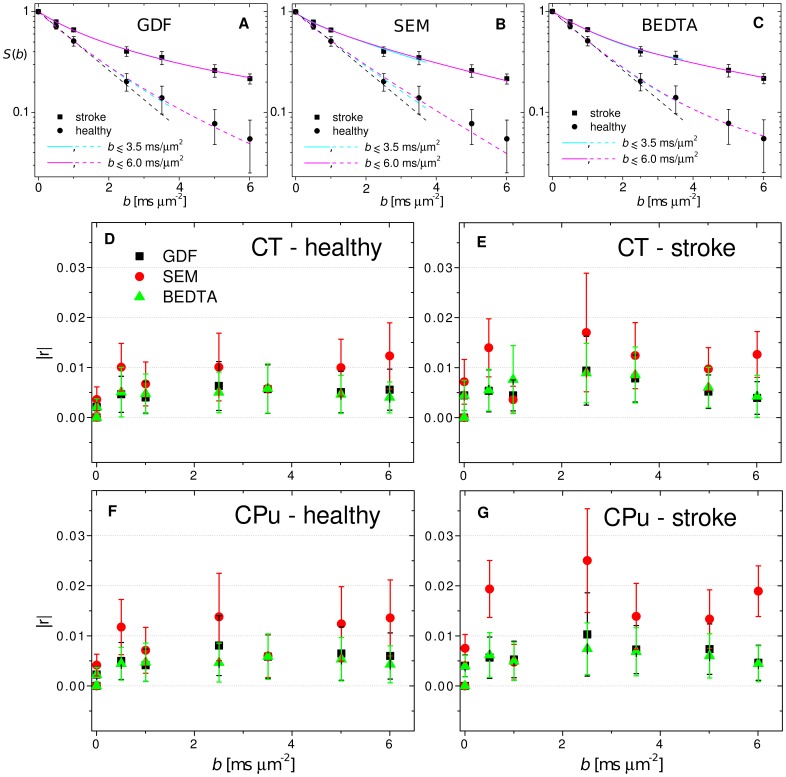
Experimental DW signal, fits and residuals. Diffusion-weighted signal as a function of *b*-value for two selected voxels in the ischemic and healthy regions. The fits (A, B, C) are shown for the gamma-distribution function (A), the stretched-exponential model (B), and the biexponential diffusion tensor analysis (C) for two fitting ranges. Absolute residuals (D–G) as a function of *b*. The bars indicate standard deviations around the means.

**Figure 3:**
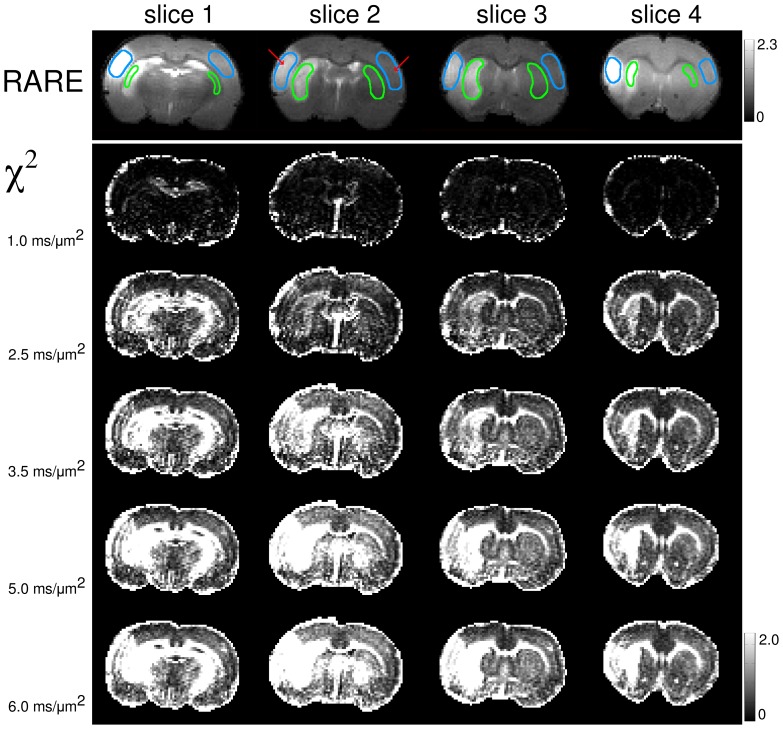
Dependence of the χ^2^-maps on the fitting range. Anatomical RARE (Rapid Acquisition with Refocused Echoes) images (upper panel) and χ^2^-maps (lower panel) for various *b*-values and four slices in animal 1. The errors increase throughout the image with increasing *b*. In lesions and white matter regions the increase is especially strong, leading to a more clear contrast at larger *b*. The regions-of-interest used for averaging of the measured parameters are shown in RARE images in cyan and green. Red arrows indicate the locations of the voxels used in Figure 2.

Typical diffusion attenuation curves for two representative voxels located in healthy and affected regions are shown in Figures 2a–2c, together with their fits using GDF, SEM and BEDTA, respectively. Fit parameters are listed in Table 1. The monoexponential function is shown as a black dashed line for the healthy voxel as an example. Clear deviations from the monoexponential behaviour occur for *b*>1 µm^−2^ ms. In Figure 3, χ^2^-maps of the monoexponential fits visualise how these deviations increase with increasing *b*-value. The maps are shown for 4 different slices in animal 1. At *b* = 1 µm^−2^ ms, the χ^2^-maps look rather homogeneous but exhibit increasing contrast between GM and WM (see the “brightening” of WM tracts such as the corpus callosum) in accordance with similar findings in human brain [68]. Moreover, stroke lesions cannot be recognised in χ^2^-maps for *b*≤1 µm^−2^ ms (see anatomic RARE-maps for their locations) but become strikingly enhanced for larger *b*. Thus, the χ^2^-maps provide a clear evidence for that the degree of non-Gaussianity is higher in stroke lesions than in healthy tissue.

**Table 1:** The values of fit parameters for GDF (*θ*, *σ*), SEM (DDC, *α*_SE_), and BEDTA (ADC_f_, ADC_s_, and *f*_f_) in two fitting ranges; the values of 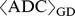 and *σ*_GD_ correspond to fit parameters of GDF.

		*b* = 3.5 ms µm^−2^		*b* = 6.0 ms µm^−2^	
model	parameter	healthy	stroke	healthy	stroke
GDF	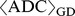	0.71±0.02	0.52±0.03	0.72±0.02	0.51±0.02
	*σ*_GD_	0.30±0.05	0.49±0.06	0.35±0.04	0.47±0.04
	*θ*	0.13±0.04	0.46±0.12	0.17±0.04	0.43±0.08
	*κ*	5.60±1.89	1.13±0.30	4.23±0.99	1.18±0.22
SEM	DDC	0.63±0.01	0.32±0.01	0.62±0.02	0.31±0.01
	*α*_SE_	0.92±0.03	0.78±0.04	0.89±0.03	0.74±0.03
BEDTA	ADC_f_	0.74±0.01	0.66±0.19	0.77±0.05	0.89±0.15
	ADC_s_	0.01±0.49	0.01±0.19	0.15±0.01	0.15±0.03
	*f*_f_	0.93±0.14	0.72±0.26	0.88±0.08	0.46±0.09

The experimental curves together with their fits are shown in Figure 2. All diffusivity values are in ms^−1^ µm^2^.

**Figure 4:**
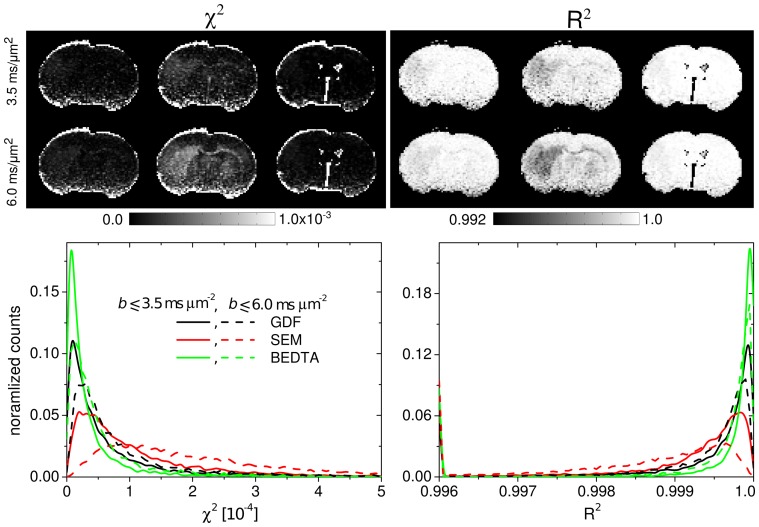
Estimates of the goodness-of-fits. χ^2^- and R^2^-maps and the corresponding histograms in ranges 1 and 2.

The fit values (see Table 1) were similar for both ranges of *b*-values with the exception of ADC_s_ in BEDTA. The overall fit quality by all three models was good in both fitting ranges as demonstrated in Figure 4 by the corresponding χ^2^- and R^2^-maps and their histograms averaged over all slices in animal 1, as an example, as well as in Figures 2d–2g by the analysis of the mean absolute residuals as a function of *b*-value. In both ranges, the χ^2^- and R^2^-maps look similar to each other for all three models demonstrating a satisfactory homogeneity across most parts of images with rather low values for χ^2^, and values close to 1 for R^2^. Somewhat increased lesion-to-tissue and WM-GM contrasts can be recognised for SEM in range 2 (when comparing to range 1 and to other models), indicating larger errors. A more detailed statistical analysis is represented by the values of 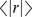 evaluated separately for the four ROIs studied (CT healthy, CT lesion, CPu healthy, CPu lesion) and averaged over all slices in animal 1. The mean residuals exhibited no tendency for systematically increasing errors with increasing *b*-values, indicating robustness of the fits regarding the range. At any *b*-values, the values of 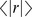 did not exceed approximately ±0.02 for GDF and BEDTA, and ±0.04 for SEM. SEM shows, in general, larger errors than GDF and BEDTA. However, based on the analysis of the residuals and of standard deviations of the fitted values, both the GDF and SEM models provided satisfactory fits. For BEDTA, a satisfactory reliability of the fitted parameters was found only in the range 2, whereas the standard deviations of the fitted parameters in range 1 were too high, see Table 1. This is because, in range 1, the DW signal was dominated by the fast component, and the slow component could not be reliably estimated. Therefore, we omitted the results of the statistical analysis for BEDTA in range 1 from further consideration.

**Figure 5:**
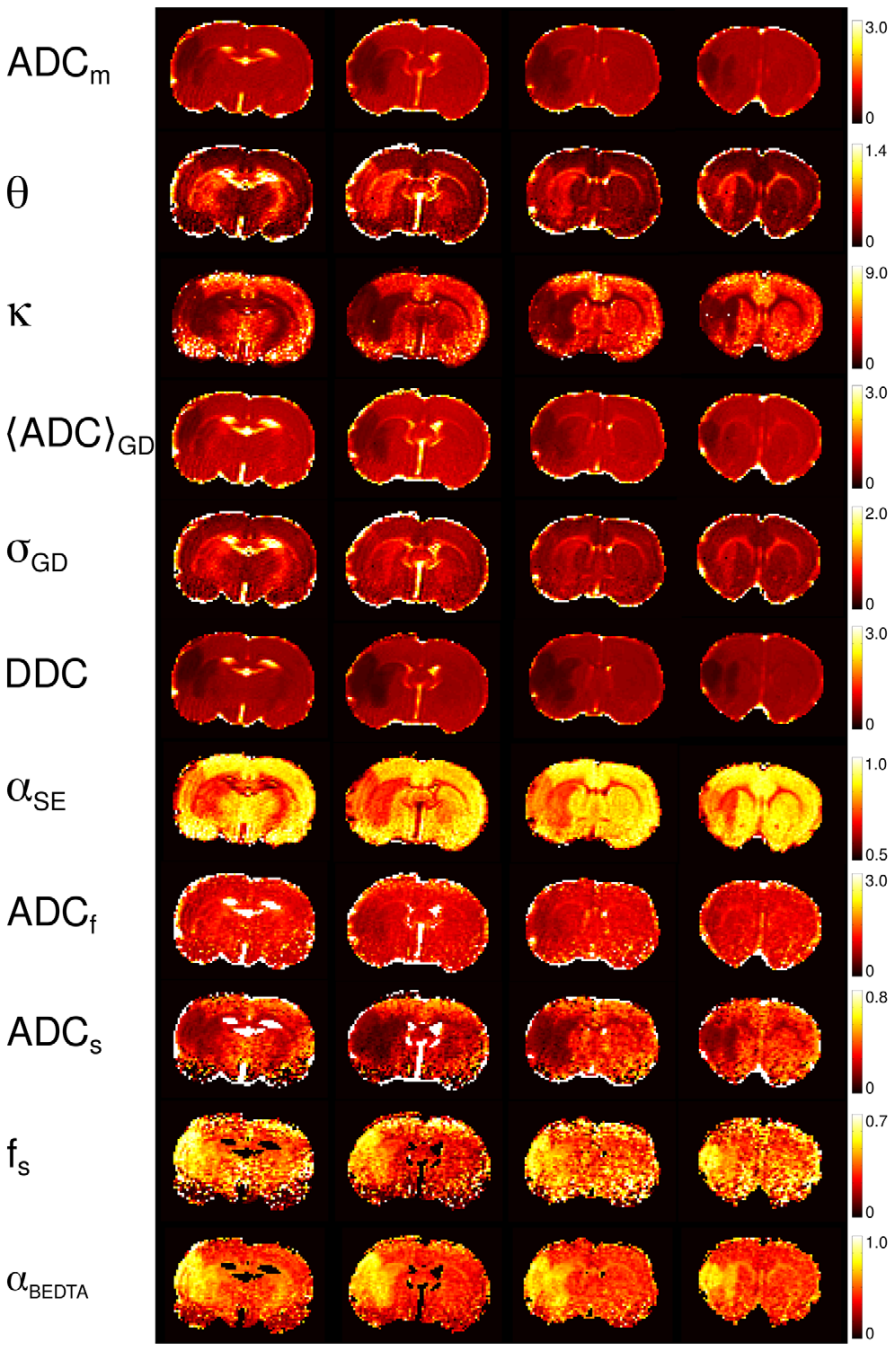
Parameter maps (animal 1). The maps of the investigated model parameters of the four slices in animal 1. The numbers on the scale bars that refer to ADC_m_, *θ*, 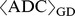, DDC, ADC_f_, and ADC_s_, are in units ms^−1^ µm^2^.

Figure 5 shows the maps of ADC_m_, *θ*, *κ*, 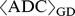, *σ*_GD_, DDC, *α*_SE_, ADC_f_, ADC_s_, *f*_s_, and *α*_BEDTA_ for animal 1, all of them providing a clear contrast for two of the ischemic lesions. These maps refer to the fitting range 2. The values of ADC_m_, *κ*, 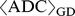, DDC and *α*_SE_, ADC_s_, and *f*_s_ and *α*_BEDTA_ were strongly decreased in the ischemic regions, whereas *θ* and *σ*_GD_ were significantly enhanced. The t-tests performed on these values, comparing healthy against affected ROIs, showed that the changes in all above parameters were significant (p<0.001).

The contrasts provided by the various model parameters exhibit essential differences. When comparing them to the conventional ADC_m_-map, the most similar contrast was produced by 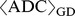. This is not surprising since ADC_m_, evaluated in the low *b*-value range must be rather close to the mean diffusivity represented by 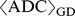, see Eq. (5). This finding confirms the robustness of the GDF model: the values of 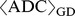 were directly evaluated from the fitted values of *θ* and *κ* using the large range of *b*-values, up to 6 µm^−2^ ms, where the signal was reduced by a factor 5 to 10. The DDC-maps provided a somewhat greater contrast than the ADC_m_-maps. Considering BEDTA, we may state that the corresponding parameter maps were much noisier than the maps of the other models. Nevertheless, the BEDTA-maps provided useful information showing that, in stroke, ADC_s_ decreases more than ADC_f_, and that *f*_s_ increases. This emphasises that the dominant changes in diffusion are associated with the slow diffusion component. *α*_BEDTA_-maps produced much more clear visualization of the lesions than each of the three fitting parameters taken separately.

**Figure 6:**
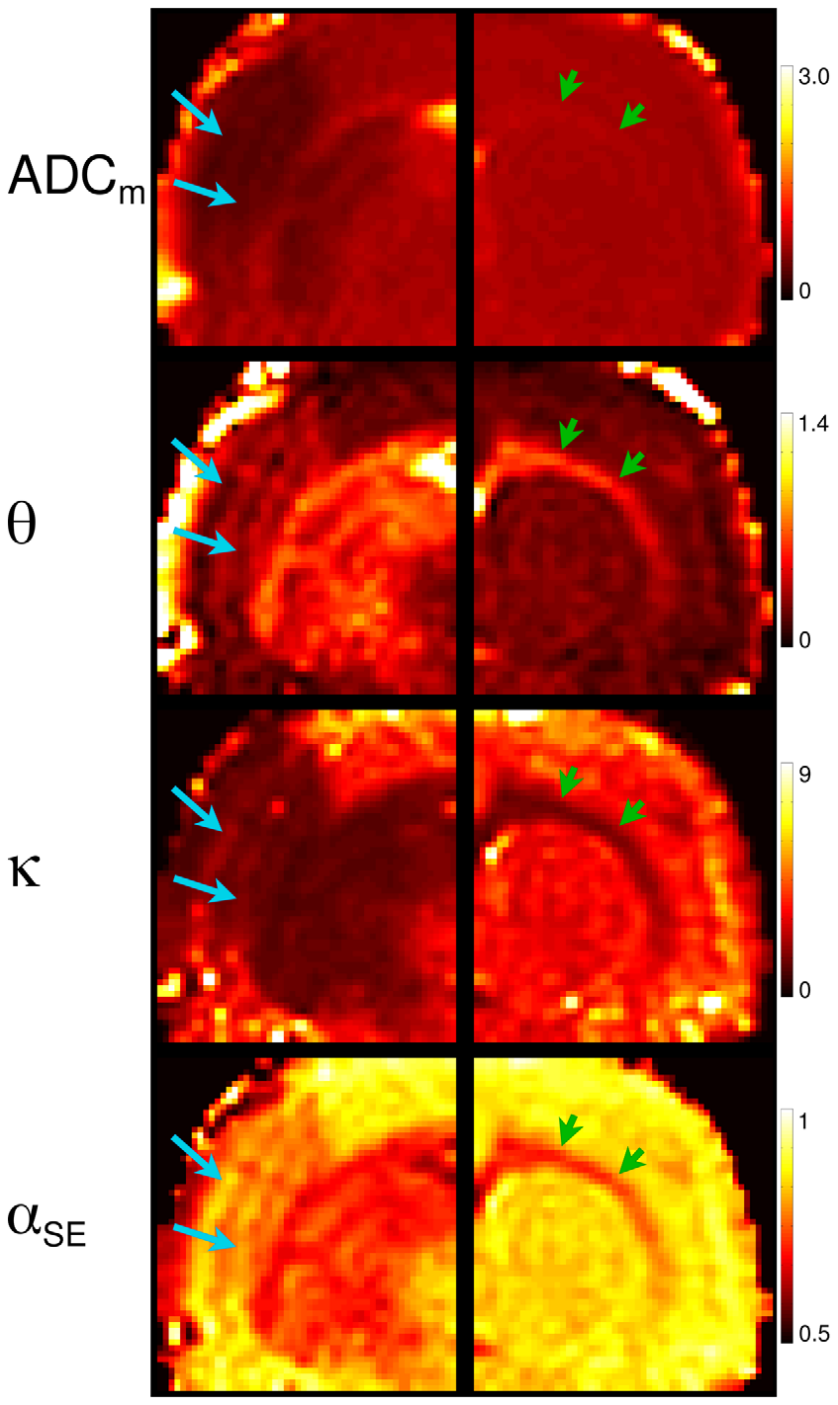
Zoomed regions. Zoomed regions of the maps shown in Figure 5 for selected parameters best representing the layered structure in the lesions (left, slice 1) and WM tracts (right, slice 3).

**Figure 7:**
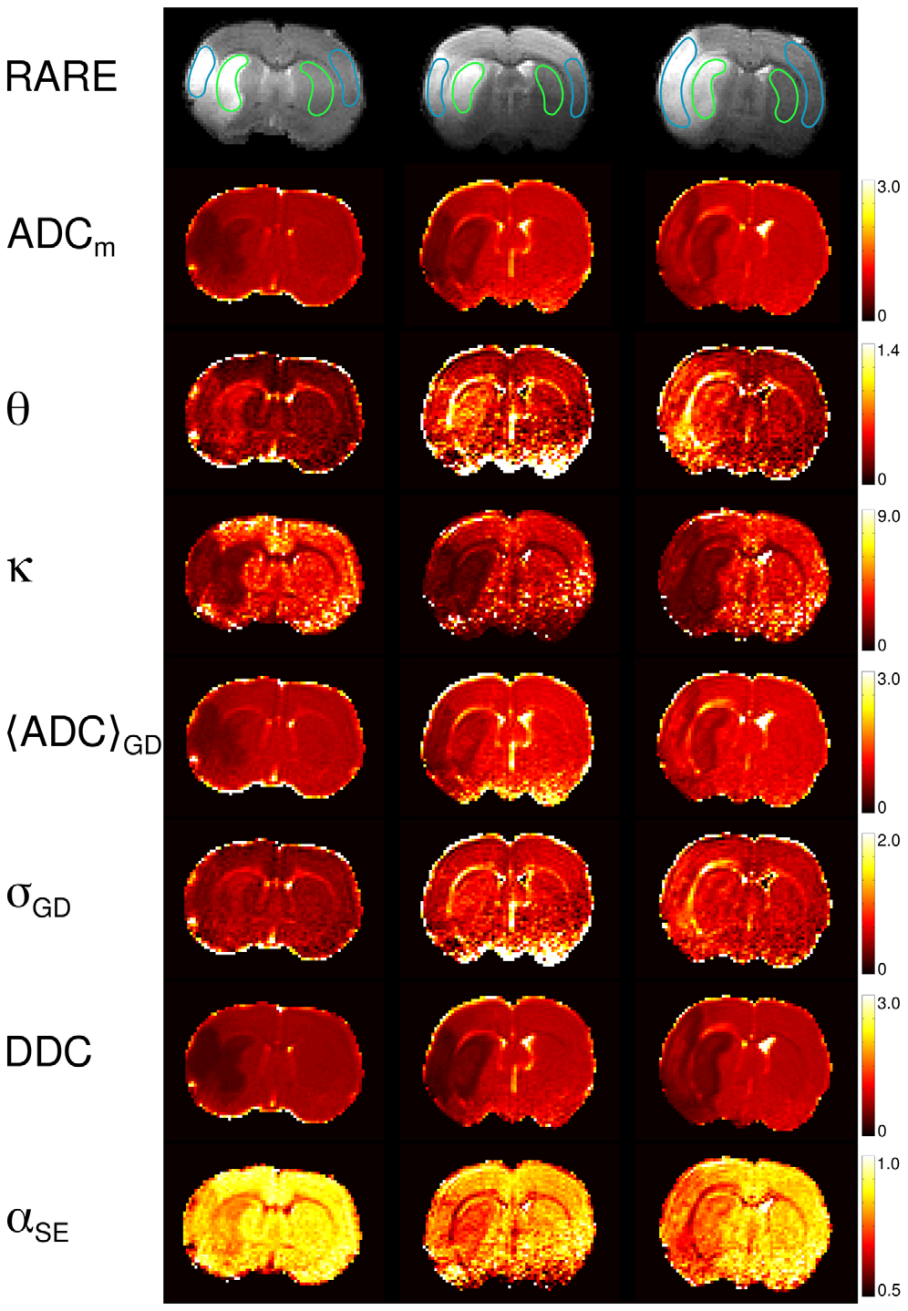
Parameter maps (all animals). Anatomical RARE ((Rapid Acquisition with Refocused Echoes)) images and maps of ADC_m_, *θ*, *κ*, 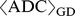, *σ*_GD_, DDC, *α*_SE_ for one selected slice best representing the ischemic lesion in each of the three animals. All diffusivities are in units ms^−1^µm^2^. The regions-of-interest used for averaging of the measured parameters are shown in RARE images in cyan and green.

In several maps of non-Gaussian quantities, *θ*, *κ*, *α*_SE_ and *α*_BEDTA_, an apparent stratified laminar structure of the cortex can be observed in the lesions whereas it is difficult to be recognised in the ADC_m_-maps. This is demonstrated in detail by the zoomed areas of the selected parameters in Figure 6, left column. The laminar structure is best visualised in the *α*_SE_ -map. Interestingly, it is apparent only in the stroke-affected tissue providing evidence for differences in the ischemic response by different cortical layers. In general, the non-Gaussian parameters tended to capture microstructural features in more detail than the diffusivity maps: see WM tracts that appear much brighter in *θ*-, *κ*-, and *α*_SE_-maps than in the ADC_m_-maps in Figures 5 and 6 (zoomed areas, right panel). These effects remained when fitting the data over the smaller range of *b*-values, *b*≤3.5 µm^−2^ ms, as demonstrated in Figure 7 for one selected slice in each of the animals. Here also the structural details were more favourably visualised in the *θ*-, *κ*- , *σ*_GD_- and *α*_SE_ -maps rather than in the ADC_m_-, 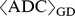
- or DDC-maps.

**Figure 8:**
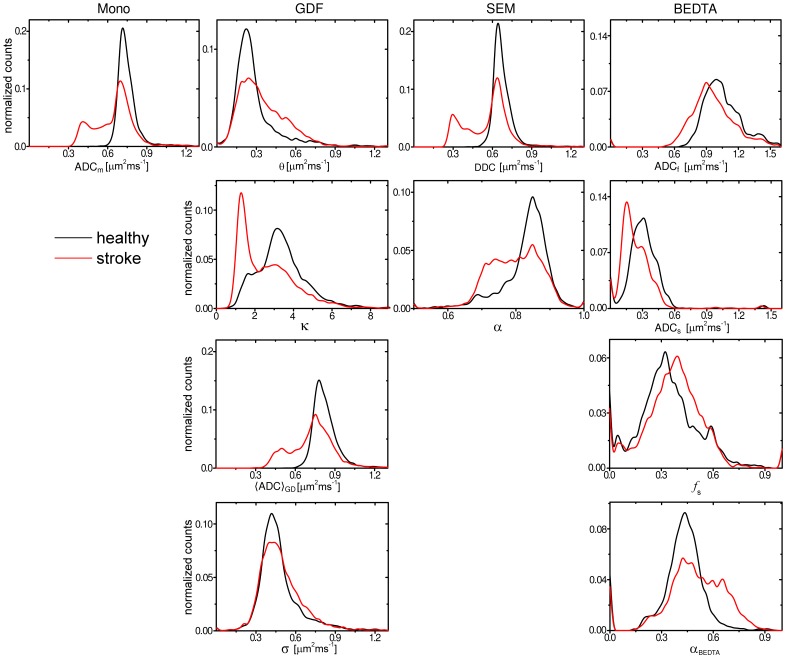
Histograms of parameter metrics. Histograms of parameter metrics of the investigated models evaluated separately for affected and contralateral hemispheres taken over all slices (animal 1).

The observed contrasts were accompanied by significant shifts of the parameter histograms shown in Figure 8. The histograms of diffusivities, ADC_m_, 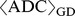 and DDC, exhibit similar shifts of their substantial parts towards significantly smaller values. The histograms of non-Gaussianity metrics, *κ* and *α*_SE_, were also shifted towards smaller values with a clear peak-like feature in the *κ*-histogram compared to a shoulder-like spread in *α*_SE_. Shifts towards higher values were observed in the histograms of *θ* and *f*_s_.

Table 2 summarises the average values of various metrics as well as their relative changes in the affected versus healthy regions (all parameters refer to animal 1, range 2). When comparing two models, GDF and SEM, the largest changes were observed for *θ* in GDF (75% in CT and 61% in CPu) followed by *κ* (−65% in CT and −57% in CPu). The changes of 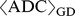 (−38% in CT and −28% in CPu) were similar to that of ADC_m_ (−42% in CT and −32% in CPu). SEM exhibited larger changes of DDC (−51% in CT and −42% in CPu) than ADC_m_. However, the relative changes in α_SE_ (about 8%) were low compared to other metrics. BEDTA parameters exhibited a larger decrease of ADC_s_ (−54% in CT and −56% in CPu) relative to both ADC_f_ (−18% in CT and −20% in CPu) and ADC_m_.

**Table 2:** Relative changes of the model parameters evaluated in two lesions, CT and CPu, averaged for 4 slices in animal 1.

	CT			CPu		
	Healthy	Stroke	Relative change [%]	Healthy	Stroke	Relative change [%]
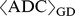	0.79±0.10	0.49±0.11	−38	0.79±0.05	0.56±0.07	−28
*σ*_GD_	0.41±0.11	0.43±0.14	5	0.45±0.07	0.49±0.08	8
*θ*	0.21±0.09	0.37±0.17	75	0.26±0.07	0.42±0.09	61
*κ*	4.14±1.94	1.43±0.32	−65	3.23±0.79	1.38±0.21	−57
DDC	0.67±0.07	0.33±0.04	−51	0.65±0.03	0.37±0.04	−42
*α*_SE_	0.88±0.03	0.81±0.05	−7.9	0.86±0.03	0.79±0.03	−7.6
ADC_f_	1.05±0.25	0.86±0.24	−18	1.06±0.17	0.85±0.10	−20
ADC_s_	0.32±0.10	0.15±0.03	−54	0.30±0.07	0.13±0.02	−56
*f*_f_	0.64±0.14	0.47±0.07	−26	0.63±0.11	0.57±0.05	−9.4
*α*	0.44±0.08	0.73±0.09	65	0.53±0.09	0.64±0.07	20

All diffusivity values are in ms^−1^ µm^2^.

**Figure 9:**
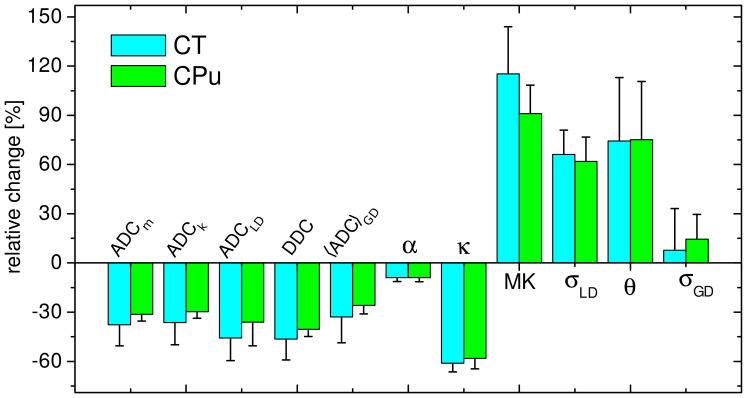
Overview of the average parameter changes. Relative changes in per cent of the values of the investigated model parameters averaged over all slices in each animal and over all three animals; error bars indicate the standard deviations. For a better overview, we included here the data for DKI (*D*_m_ and MK) and LNDFI (*D*_LD_ and *σ*_LD_) investigated in the previous work [41].

Figure 9 summarises the relative parameter changes in GDF and SEM averaged over all slices measured for all three animals (four slices in each). For a better visual comparison of the new (GDF and SEM) and old (DKI and LNDFI) models, the data is complimented by the relative changes observed for parameters ADC_K_, MK, ADC_LD_, and *σ*_LD_ investigated in the previous work [41]. Most of the parameters tend to show somewhat larger changes in CT than in CPu. All data refer to range 1. It should be noted that the larger range would be inapplicable for DKI. On the contrary, BEDTA requires a larger range of *b*-values and, therefore, is not included in this overview. Figure 9 shows that both non-Gaussian models, GDF and SEM, are more sensitive to lesions than conventional diffusion MRI. Amongst the two models, GDF and SEM, better sensitivity was provided by GDF: the parameters *θ* and κ exhibited relative changes of ∼75% and ∼−60%, respectively, versus −(40–46)% for DDC and ∼9% in *α*_SE_. However, a comparison between four models (GDF, SEM, DKI and LNDF) has shown that DKI provides the largest sensitivity followed by GDF, LNDF and SEM.

**Figure 10:**
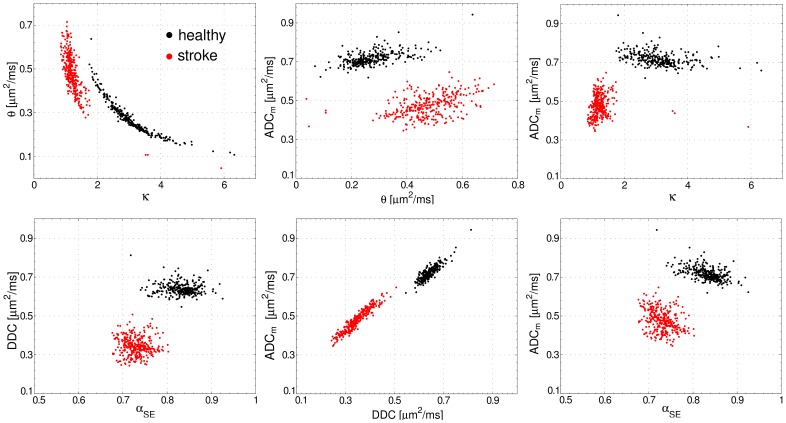
Scatter plots. Scatter plots for different combinations of parameters: *θ* vs. *κ* (gamma-distribution function, GDF) DDC vs. *α*_SE_ (stretched exponential model, SEM), and ADC_m_ vs. each of the GDF and SEM parameters. The Pearson's, *r*, and Spearman's, *ρ*, correlation coefficients are shown in Table 3.

Figure 10 demonstrates the scatter plots for the model parameters, *θ* and *κ*, and DDC versus *α*_SE_, as well as between ADC_m_ and θ, *κ*, DDC or *α*_SE_, for ROIs in lesions and contralateral tissue. The data refer to animal 1 and all slices. One can clearly see that the scatter plots allow one to separate the voxels belonging to the affected and healthy tissue with practically no overlap. The corresponding Pearson's, *r*, and Spearman's, *ρ*, correlation coefficients are represented in Table 3 for completeness. However, more detailed analysis of these coefficients was outside the focus of this paper.

**Table 3:** The Pearson's, *r*, and Spearman's, *ρ*, correlation coefficients for the data shown in Figure 10.

		GDF			SEM		
		*θ* vs. *κ*	ADC_m_ vs. *θ*	ADC_m_ vs. *κ*	DDC vs. *α*_SE_	ADC_m_ vs. DDC	ADC_m_ vs. *α*_SE_
*r*	healthy	−0.89	0.58	−0.40	−0.09	0.88	−0.54
	stroke	−0.70	0.47	0.26	−0.04	0.95	−0.33
*ρ*	healthy	−0.99	0.56	−0.44	−0.06	0.83	−0.53
	stroke	−0.71	0.46	0.25	−0.08	0.95	−0.34

All correlations were statistically significant (*p*<0.001) except for DDC vs. *α*_SE_.

## Discussions and Conclusion

Development of non-Gaussian diffusion imaging is emerging as a novel tool providing clinically useful information on brain tissue. Related to stroke, investigation of non-Gaussian models has a very short history represented by a few papers published during the last couple of years [9], [10], [41]–[43]. These works have shown promising potentials in using those models for stroke assessment. In the present work we examined three additional non-Gaussian approaches, SEM, GDF and BEDTA. The two former models have not been previously applied to stroke. Moreover, while GDF was used to characterise the distribution of axon diameters [34] and of macromolecular diffusivities in polymers [54], it has not been used to model the diffusion response in the brain tissue. We compared the results with the conventional monoexponential model and two previously investigated models, DKI and LNDFI, using the same data sets. To date, few reports exist in which several non-Gaussian models are compared on the same data sets of *in vivo* [36], [69] or *in vitro* [70] experiments. The emphasis of those studies was put more on the methodological issues such as goodness-of-fit, influence of noise, or model ranking. While we also made an estimations of the fit quality, our main interest was focused on the comparison of the performance of the non-Gaussian metrics as biomarkers of the pathological state. Given the effect of ischemic events on the tissue microstructure and diffusion properties, the animal stroke model is well suited for a comparison of various methods. Similar to DKI and LNDFI, the GDF and SEM models demand a minimum of 3 *b*-values so that, with no extra time for measurements, all parameters of these 4 models can be obtained in post-processing based on the same data. We assume that our comparative investigation should contribute to further development and improvement of diagnostic tools in stroke.

Ideally, one would like to fit the experimental curves over the largest *b*-value range possible, to yield maximal attenuation of the signal from its initial value. However, various factors limit the usable *b*-value range, with noise being the most typical problem at high *b*-values. An applicability of each model is, in turn, another important issue. As well-known examples, DTI is limited to the low *b*-values range (*b*≤1 µm^−2^ ms), whereas DKI can be applied to more extended but also limited (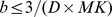) range. The applicability of DKI in our work corresponds to the range 1. If the applicability range is limited, more caution is required when comparing results obtained by different research groups since the evaluated metrics and their variation may significantly depend on the selected range of *b*. Although a detailed analysis of the *b*-value dependence of fitting parameters was beyond the scope of this work, we checked the applicability of the investigated models in two fitting ranges, moderate and extended. GDF and SEM, to their advantage, occurred to be relatively robust with respect to the selected range of *b*-values. The same applies also to LNDFI studied in the previous work [41]. BEDTA, on the other hand, was applicable only in range 2. In comparison to the other investigated models, BEDTA is less robust as it contains one more fitting parameter (3, if signal intensities are normalised), and is more vulnerable to low signal-to-noise ratio [36], [68]. Therefore, it is less beneficial for stroke analysis as it demands more scanning time. Nevertheless, application of BEDTA allowed us to receive additional useful information as it directly showed that the dominant contribution to the reduction of the mean diffusivity is due to the slow diffusion component and includes both the decrease of ADC_s_ and increase of *f*_s_. An increase of *f*_s_ in ischemic human brain was reported also in Ref. [71], although the authors observed an increase rather than a decrease of ADC_s_. In ouabain induced cell swelling in a perfused rat hippocampal slice, Buckley et al. [72] also observed an increase of *f*_s_, however, not accompanied with any significant changes in the values of ADC_f_ and ADC_s_. Based on observations related to biexponential behaviour, Le Bihan et al. [5], [15] emphasized the dominant role of membranes, bound water and the residence times near tissue boundaries affecting the diffusion behaviour in stroke.

Several parameters map studied in this work were able to provide a better visual contrast than the conventional ADC_m_ map and exhibited larger relative changes in ischemic versus healthy tissues. The smallest change was detected for α_SE_ in SEM. In contrast, the DDC of this model provided a somewhat larger change than ADC_m_. Analogously, smaller sensitivity of the stretched exponent, *α*_SE_, in comparison to other non-Gaussian parameters, was observed also as a function of fibre density, which modulates the tortuosity of the interstitial space, in the physical diffusion fibre phantoms [73]. A cancer study using SEM has shown that, in tumours, *α*_SE_ had similar values to that in WM but much lower values than the typical tumours in GM [49]. In general, based on the amount of the average change over all slices and animals, the parameters of the four models (GDF, SEM, DKI, and LNDFI), see Figure 9, demonstrate that the largest changes were observed in MK, followed by *θ*, *κ*, *σ*_LD_, DDC, ADC_LD_, ADC_K_, ADC_m_, and, finally, by *α*_SEM_. The non-Gaussianity metrics (with the exception of *α*_SE_) provided a spectacular gain in contrast relative to the “gold standard”, ADC_m_.

More detailed information can be gathered from the analysis of the histograms and scatter plots. The histograms of various parameters exhibited significant differences in their shapes that can explain the different sensitivities to stroke of the mean values. In particular, the histogram of DDC exhibits a splitting into two distinguished peaks similar to that of ADC_m_ (attributed to the healthy and affected areas). In contrast, the histogram of *α*_SE_ shows a continuous broadening towards the lower values. Although the associated change of the mean is not very large, we see that a significant part of the histogram does not overlap with that of the healthy counterpart. This can explain an observed high laminar contrast in lesions produced by *α*_SE_-maps although the sensitivity of the mean value was low.

In most of the studies, the model parameters were usually analysed separately from each other. At the same time, their simultaneous changes may be more informative. We demonstrated that ischemic lesions tend to be well separated from the healthy tissues using the correlation plots. We expect this kind analysis to be helpful in diagnostics, as it should allow one to increase the certainty and reliability of the pathological state assessment. The scatter plots may also be valuable for post-stroke monitoring of the patients as, with time after onset, the diffusivity values begin to normalise and do not provide clear characteristics for the tissue state. The same approach can be useful regarding other pathologies, especially if they are related to GM. This is because DTI metrics important in WM, such as FA and axial or radial diffusivities, are not predicative in GM due to its isotropy. Non-Gaussianity parameters, in contrast, are thought to quantify the complexity of the tissue microstructure rather than its anisotropy.

An interesting finding of this work is the appearance of laminar cortical structures in stroke lesions. The genuine differences in cortical layer microstructure are well-known from histology, as shown in Figure 1. However, observation of cortical layers with MRI is a challenging task [74]–[76] and requires special efforts, such as using manganese-enhanced methods [75]. In healthy regions, no clear laminar contrast was observed but became distinguishable in the lesions represented via *α*_SE_- , *θ*- and *κ*-maps. Retrospectively, the laminar structure can also be recognised in *σ*_LD_-maps of LNDFI shown in Figures 2 and 4 of the previous work [41]. However, it is hardly visible in the diffusivity maps. Our finding allows us to propose that the cascade of ischemic processes tends to non-uniformly affect the cortical layers differentiating by their cyto- and myeloarchitecture [74]. Based on diffusion studies, such phenomenon has not been reported before. However, a significant difference between cortical layers in the time profile of eosinophilic neurons in the post-ischemic cortex was reported by Sun et al. [77]. Furthermore, the layered structure as a response to stroke was observed in MRI images one week after MCAO [78]. Sbarbati et al. [78] observed three layers and interpreted their origin due to enlargement of the pial space in the external layer, degenerating nervous tissue accompanied by a massive accumulation of macrophages in the middle layer, and by the presence of edematous nerve tissue without a marked accumulation of macrophages in the deepest layer. In our study, in difference, a stratified structure was observed only 24 hours after stroke. Selective vulnerability of cortical layers to ischemia can also be due to higher metabolic demand and denser concentration of receptors for excitatory amino acids [79]. In this context, it should be noted that the larger parameter changes observed in CT (GM) than in CPu (mixture of GM and WM) might also be due to microstructural differences in these anatomical regions and different susceptibility of GM and WM to ischemic injury. However, elucidation of the underlying mechanisms requires a special focus in a dedicated future work, and was beyond the scope of this paper.

Understanding of how ischemic processes are related with the fine microstructural features could greatly improve our knowledge of the biophysical mechanisms of the ischemic damage [80]. These mechanisms have not been understood in detail so far and remain a subject of debate. Swollen cells, increased tortuosity of the ECS, changes in membrane permeability, increases in the amount of bound water and, proposed more recently, neurite beading may all come into play [81]. In this context, non-Gaussian metrics provide complementary data to infer valuable microstructural information for comparison with existing models of stroke. Useful models should be able to explain not only the average decrease of the mean diffusivity by about 40% but also a simultaneous large increase of deviations from the Gaussian model as quantified above. For example, increasing tortuosity of the ECS alone is not likely to be sufficient to explain the amount of the observed changes. This consideration is based on a comparison of our results with those obtained for anisotropic fibre phantoms [73], where the tortuosity was modulated by the fibre density. It was shown that changing fibre density from 0.45 to 0.7 (close packing density) is accompanied by a three-fold decrease of the diffusivity whereas kurtosis and *σ*_LD_ have increased by rather moderate 50%. Qualitatively, our results would be compatible with the model of cell swelling accompanied by neurite beading already suggested to explain observed increases in kurtosis in human stroke [9], [10]. This model would non-uniformly increase the contribution of slow diffusion fractions by more restricted environments both in the ICS and ECS. Also increases in the amount of bound water or a reduction in the membrane permeability could lead to an increase in the slow diffusion fraction. More work is required to understand the complex cascade of pathological processes in stroke that change an environmental landscape for diffusing water molecules. Among others, one needs to perform the studies with a large group of animals in order to reduce inter-subject variability and enhance a statistical significance. However, with further theoretical and experimental developments, these biophysical changes can be expected to be better quantified with non-Gaussianity metrics.

In conclusion, we have shown that non-Gaussian metrics are useful in the elucidation of stroke and provide potentially valuable information. This refers, in particular, to enhanced contrast of the lesions and its quantification with respect to healthy tissue and to the fine microstructure differences within the lesions, such as the observation of cortical laminar structures. Two-dimensional scatter plots allow one to delineate affected tissue with better reliability.
